# Methods for analyzing longitudinal data from randomized pretest-posttest-follow-up trials in behavioral research: a practical guide to latent change models

**DOI:** 10.1007/s10865-025-00600-y

**Published:** 2025-09-09

**Authors:** Constance A. Mara

**Affiliations:** Behavioral Medicine and Clinical Psychology, Cincinnati Children’s Hospital Medical Center, Department of Pediatrics, University of Cincinnati College of Medicine, 3333 Burnet Avenue, MLC 7039, Cincinnati, OH 45229 USA

**Keywords:** Randomized trials, Longitudinal data analysis, Behavioral interventions, Latent change models

## Abstract

Randomized pretest, posttest, follow-up (RPPF) designs are widely used in longitudinal behavioral intervention research to evaluate the efficacy of treatments over time. These designs typically involve random assignment of participants to treatment and control conditions, with assessments conducted at baseline, immediately post-intervention, and during the follow-up period. Researchers primarily focus on determining whether the intervention is more effective than the control condition at post-treatment and whether these effects are sustained or change over time. This paper presents Latent Change Models (LCMs) as a practical approach for analyzing randomized pretest–posttest–follow-up (RPPF) trials, directly estimating discrete changes between timepoints and intervention–control group differences. The utility of LCMs is demonstrated through an application to the STAR (Supporting Treatment Adherence Regimens) trial, a pediatric randomized behavioral clinical trial aimed at improving adherence to anti-seizure medications (ASMs) among children with new-onset epilepsy. The results of the trial analyzed via an LCM are contrasted with the results as analyzed by an ANCOVA, a longitudinal linear mixed-effects model, and a latent growth curve model. The tutorial and application to the STAR trial demonstrate that LCMs offer notable strengths, including the ability to estimate discrete changes over time, control for baseline variability in the outcome, and incorporate all longitudinal data within a single, parsimonious model. These models provide an accurate and nuanced understanding of intervention effects in RPPF designs, with implications for clinical intervention research.

**Supplementary Information:**

The online version contains supplementary material available at 10.1007/s10865-025-00600-y.

## Introduction

Randomized pretest, posttest, follow-up (RPPF) designs are a foundational methodology in behavioral intervention research, offering a robust framework to assess changes in outcomes or intervention targets over time. These designs involve random assignment to treatment or control conditions, with outcomes measured at several critical timepoints: pre-intervention (pretest), immediately following the intervention (posttest), and after a delay (one or more follow-ups). This structure provides a comprehensive understanding of intervention efficacy and long-term sustainability, typically addressing two questions central to behavioral intervention research: (1) Do the treatment and control groups differ in the amount of change from pretest to posttest? and (2) Do the treatment and control groups differ in the amount of change from posttest to follow-up(s)? By capturing both immediate and delayed outcomes, RPPF designs are instrumental in evaluating interventions aimed at enduring behavior change, such as psychotherapy for mental health disorders, substance use prevention, or weight-loss programs (Collins et al., 2009; Kazdin, 2003).

The importance of RPPF designs lies in their capacity to assess trajectories of change. For example, in clinical psychology, a cognitive-behavioral therapy (CBT) intervention may significantly reduce symptoms of depression at posttest, but only through follow-up assessments can researchers determine whether these gains are maintained or if relapse occurs (Cuijpers et al., 2020). Similarly, in behavioral medicine, interventions targeting lifestyle changes, such as dietary improvements or smoking cessation, often rely on RPPF designs to evaluate both short-term adherence and long-term health outcomes (Prochaska & Velicer, 1997).

Numerous statistical methods have been developed to evaluate change over time, either within or between groups, such as paired-samples t-tests, analysis of variance (ANOVA), analysis of covariance (ANCOVA), multivariate analysis of covariance (MANCOVA), and multiple regression. More advanced techniques, like latent growth curve modeling (LGCM) and longitudinal mixed-effects modeling (LMM), offer enhanced approaches for analyzing change by leveraging all available longitudinal data in a randomized pretest–posttest-follow-up (RPPF) design within a unified model, thereby increasing statistical power (Bryk & Raudenbush, 1987; Meredith & Tisak, 1990). In the context of RPPF designs, the choice of method should align with the study’s goals, assumptions, and the structure of change over time. For example, ANCOVA remains widely used in behavioral intervention trials, particularly when focusing on differences at a single posttest timepoint (e.g., Cuijpers, et al., 2020), and it is especially useful for isolating treatment effects while adjusting for baseline variability. However, this approach does not fully leverage the repeated-measures nature of RPPF designs (Rausch et al., 2003). Although LMMs and LGCMs are well established and flexible frameworks for modeling longitudinal data, they are often parameterized to capture continuous or smooth change over time. Without additional customization, such as spline or piecewise modeling, these approaches may be less sensitive to detecting abrupt or timepoint-specific changes, such as immediate post-intervention effects or delayed outcomes at follow-up (Mun et al., 2009; Singer & Willett, 2003). This can be a meaningful consideration in behavioral intervention research, where non-linear patterns of change and phase-specific effects are often of theoretical and clinical importance.

To support researchers in selecting appropriate analytic strategies for RPPF designs, this paper introduces Latent Change Models (LCMs) as a flexible and accessible option that may be less familiar to some applied behavioral scientists. LCMs offer a framework that explicitly models change between specific timepoints, allowing for nuanced examination of intervention effects at multiple phases of a study. These models enable researchers to estimate discrete changes, evaluate the magnitude and direction of change, and examine individual variability in change trajectories within a single model (McArdle, 2009; Mara et al., 2012; Mun et al., 2009). This tutorial provides a detailed overview of LCMs, their application in behavioral clinical trials, and practical implementation using data from the Supporting Treatment Adherence Regimens (STAR) trial—a randomized intervention designed to improve treatment adherence in children with epilepsy (Modi et al., 2021). To orient readers to the unique structure and capabilities of LCMs, we compare results from LCMs to those obtained using more commonly applied methods (ANCOVA, LMM, LGCM), highlighting how different parameterizations can yield distinct insights about intervention effects in RPPF designs.

### Software note

This tutorial will present code in Mplus to implement the LCM models discussed (see Appendix). There are several other software options—such as Stata, R, SAS, and SPSS—that can also perform the analyses described. Each software has unique strengths and weaknesses, and the choice may depend on factors such as user familiarity, specific analytic needs, and computational preferences. Readers are encouraged to utilize the software they are most comfortable with to perform their analyses. While this tutorial showcases specific Mplus code for concreteness, the principles and methods discussed are broadly applicable across other software platforms. Equivalent implementations exist in R/lavaan, OpenMx, Onyx, EQS, Stata, etc., and our equations and path diagrams are software-agnostic.

### Terminology note

There are many phrases that can be used to describe the timepoints in a trial. In this paper, pretest is defined as the assessment point prior to the intervention (also known as time 0, baseline, pre-intervention, etc.). Posttest is defined as the timepoint immediately following the intervention (also known as post-intervention, time 1, post-treatment, etc.). Follow-ups are defined as any timepoint after the posttest assessment point. Usually, there is at least one follow-up assessment point, but there can be numerous. Sometimes, these follow-ups can be the primary timepoint of interest in a trial, but are typically used to assess the sustainability of the intervention over time.

This tutorial assumes that readers have a working-level knowledge of regression, multilevel models, and latent growth curve models. For more information about ANCOVAs and regression, readers are directed to Tabachnick & Fidell, 2019, Campbell & Kenny, 1999, Huck & McLean, 1975, to name a few. For more information about multilevel models, see Bryk & Raudenbush, 1987, Gelman & Hill, 2007, and Snijders & Bosker, 2012. For more details about LGCMs, see Bollen & Curran, 2006 and Little, 2013. For readers who would like additional guidance on using Mplus software and its syntax, we refer them to the Mplus user’s guide and web page resources (Muthén & Muthén, 1998–2017).

### Analysis of covariance (ANCOVA) for RPPF designs

Analysis of covariance (ANCOVA) is a widely used method for analyzing data from randomized pretest–posttest-follow-up (RPPF) designs (e.g., Cuijpers et al., 2020; Stice et al., 2006; van Straten et al., 2008). By combining regression and analysis of variance, ANCOVA incorporates covariates such as pretest scores to adjust for pretest differences between intervention groups, increasing power. Its popularity stems from its simplicity and interpretability, but the method has both strengths and limitations, particularly in the context of RPPF designs.

One of the key strengths of ANCOVA is its ability to control for pretest differences in the outcome scores between the intervention groups that may occur by chance, despite randomization. By adjusting for this variability in pretest scores, ANCOVA enhances statistical power and reduces error variance, ensuring a more robust comparison across intervention groups (Huitema, 2011; Rausch et al., 2003). Additionally, the results are straightforward to interpret, with adjusted means providing a clear understanding of intervention effects. The method is also flexible, allowing researchers to include additional covariates such as demographic or contextual factors, which can further improve precision and reduce error variance. For studies with small sample sizes, ANCOVA can be an efficient approach, provided its assumptions are met. Furthermore, its broad implementation across major statistical software packages makes it accessible to most researchers.

While ANCOVA remains a widely used and interpretable method in behavioral trials, particularly for comparing groups at a single posttest timepoint while adjusting for baseline scores, there are important considerations when applying it to RPPF designs. The method assumes a linear relationship between the covariate and outcome, homogeneity of regression slopes across groups, and independence between the covariate and treatment condition. Violations of these assumptions can introduce bias and reduce the validity of results (Rausch et al., 2003; Tabachnick & Fidell, 2019). In longitudinal contexts, ANCOVA is typically applied separately at each follow-up timepoint, treating outcomes as independent rather than modeling within-subject correlations. While repeated measures ANOVA and mixed ANOVA are sometimes used to evaluate longitudinal change, these approaches rely on restrictive assumptions such as sphericity, equal spacing of timepoints, and complete data. Moreover, they are typically less flexible in accommodating time-varying covariates or modeling individual trajectories. Several authors have noted that repeated measures ANOVA can be viewed as a special case of multilevel modeling (Kenny & Judd, 1986; Raudenbush & Bryk, 2002), but lacks the adaptability of modern mixed-effects frameworks. As a result, longitudinal mixed-effects models (LMMs) have largely supplanted repeated measures ANOVA in contemporary behavioral research due to their ability to incorporate missing data, irregular time intervals, and individual-level variability in change.

When assumptions are met and missing data are minimal or well-managed (e.g., through multiple imputation—see Missing Data Handling section for more detail), ANCOVA offers a straightforward and statistically efficient option. It is particularly well suited for detecting group differences at specific follow-up timepoints, which aligns closely with the types of discrete change that many clinical trialists prioritize (e.g., Dennis-Tiwary et al., 2016; Stice, et al., 2006). However, for designs involving multiple follow-up assessments, potential non-linear change, or greater heterogeneity in individual patterns, more flexible approaches such as LMMs or structural equation modeling may be preferable. These methods allow researchers to capture a broader range of longitudinal dynamics and make fuller use of the data structure.

### Longitudinal mixed-effects models and latent growth curve models for RPPF designs

Longitudinal mixed-effects models (LMMs), also referred to as multilevel (MLM) or hierarchical linear models (HLM), are a robust and flexible approach for analyzing data from randomized RPPF designs (Singer & Willett, 2003). These models account for the nested structure of repeated measures within individuals, incorporating both fixed effects (e.g., intervention effects) and random effects (e.g., individual variability). LMMs are a powerful technique for modeling longitudinal data, particularly when data are incomplete or complex. One of the primary strengths of LMMs is their ability to model individual trajectories over time. By estimating fixed effects for the overall population trends and random effects to capture individual variability, LMMs can provide detailed insights into within-person changes and between-person differences. This makes them well-suited for examining intervention effects over time, including interactions between time and intervention group (e.g., Brown, Wyman, Guo, & Peña, 2013; Chorpita et al., 2017). LMMs can handle unequal spacing of timepoints and unbalanced datasets, and the ability to model non-linear trajectories and incorporate time-varying covariates further enhances the applicability of LMMs for complex longitudinal designs.

Latent growth curve modeling (LGCM) is a specialized application of structural equation modeling (SEM) that represents individual trajectories over time using latent variables. In LGCM, repeated observed measures are treated as indicators of latent growth factors, typically an intercept (initial level) and slope (rate of change), making it particularly well suited for modeling longitudinal patterns within the SEM framework (Bollen & Curran, 2006; Little, 2013; McArdle, 2009). LGCM is widely used in behavioral and clinical research, including applications to RPPF designs (e.g., Connell et al., 2006; Feil et al., 2009; Zhou et al., 2022). A key strength of LGCM is its ability to model individual differences in trajectories. By estimating growth factors (e.g., intercepts, slopes, or higher-order parameters for non-linear change), LGCM provides a comprehensive framework for understanding both overall trends and variability among individuals. This allows researchers to test predictors or moderators of growth and assess how interventions influence change over time. LGCM also integrates well with other structural equation modeling techniques, enabling complex mediation or multivariate analyses that examine how changes in one domain affect outcomes in another (e.g., Cheong et al., 2003). The flexibility of LGCM extends to modeling non-linear change, allowing for the inclusion of quadratic or piecewise growth components, which are often needed in RPPF designs (e.g., Feil, et al., 2009). Additionally, LGCM accommodates time-invariant and time-varying covariates, enabling researchers to examine the dynamic interplay between predictors and outcomes over time (e.g., McCarty et al., 2013).

Longitudinal mixed-effects models (LMMs) and latent growth curve models (LGCMs) are powerful tools for analyzing change over time in behavioral intervention trials, including those using randomized pretest–posttest–follow-up (RPPF) designs. These methods are particularly useful when the primary goal is to estimate overall trends, such as average slopes of change, and to examine how these trends differ between groups (e.g., Singer & Willett, 2003). However, in many RPPF studies, researchers are also interested in detecting discrete group differences at specific timepoints, such as immediate post-treatment effects or sustained changes at follow-up. When intervention effects are not constant over time, but instead emerge or peak at specific assessments, trajectory-based models may obscure these differences (Rausch et al., 2003). For example, a trial may show no significant group-by-time interaction in a growth model, but still reveal meaningful group differences at posttest when analyzed using a method like ANCOVA. In such cases, modeling average change alone may overlook clinically important patterns of response. LGCMs and LMMs can be adapted to test time-specific contrasts or accommodate non-linear change (e.g., via piecewise models), but this requires deliberate specification. Although piecewise LGCMs have been effectively used in some applied trials (e.g., Cheong et al., 2003; Connell & Dishion, 2006; Feil et al., 2009), these adaptations are not consistently implemented in behavioral intervention research, where linear growth remains the default in many applications (Grimm, Ram, & Estabrook, 2017). Additionally, while both LMMs and LGCMs can include baseline outcomes as covariates, this step is not always standard practice and may be omitted, potentially complicating interpretation in the presence of baseline imbalance. In contrast, methods like ANCOVA explicitly center the analysis on group differences at a target timepoint and routinely adjust for baseline, aligning well with many trialists’ core evaluation goals.

Overall, LMMs and LGCMs offer rich insights into change processes and individual variability and remain widely used and appropriate in the analysis of behavioral trial data. However, in contexts where interest lies in discrete, time-specific effects, or when modeling simplicity and baseline control are priorities, alternative approaches such as ANCOVA or the LCMs presented next may offer useful complementary perspectives.

### Latent change models for RPPF designs

Latent change models (LCMs) are a specific application of the latent change score model (LCSM) within structural equation modeling (SEM) framework. Originally developed by McArdle and Hamagami (2001), LCSMs allow researchers to estimate change as a dynamic latent construct, capturing within-person differences across timepoints while accounting for measurement error. LCMs represent a constrained version of this broader framework, typically involving a series of latent difference scores between adjacent timepoints (e.g., pretest to posttest, posttest to follow-up). This parameterization is particularly advantageous in randomized pretest–posttest-follow-up (RPPF) designs, where the goal is to evaluate intervention effects that may emerge, dissipate, or re-emerge at discrete intervals. A major strength of LCMs is their ability to isolate and evaluate changes between discrete timepoints, a critical feature for RPPF designs that include distinct intervention phases such as pretest, immediate posttest, and follow-up. LCMs are particularly well suited when treatment effects are hypothesized to emerge between specific phases rather than develop gradually over time, allowing for focused evaluation of change that aligns with clinical or theoretical milestones in the intervention. This is especially advantageous in behavioral trials where effects may be delayed, time-limited, or nonlinear (McArdle & Hamagami, 2001; Mun et al., 2009).

Both LCSMs and LCMs are estimated within the SEM framework, which allows researchers to model unobserved (latent) variables and their relationships using observed indicators (Bollen & Curran, 2006; Little, 2013). In longitudinal contexts, SEM is particularly useful because it can account for measurement error,^1^ incorporates multiple timepoints, and handles missing data through full information maximum likelihood (FIML) estimation (Enders, 2010). These strengths make SEM-based approaches especially attractive for behavioral intervention trials, which frequently involve complex longitudinal data structures and partial data from participant dropout.

In contrast to latent growth curve models (LGCMs), which are often initially parameterized to estimate continuous trajectories using latent intercepts and slopes, LCMs model change as a series of discrete latent differences. Each change score (e.g., ΔY_posttest = Y_posttest − Y_pretest) is modeled as a latent variable and can be regressed on predictors such as treatment group to test for group differences in change. This approach is especially useful when interventions are expected to have time-specific effects rather than a uniform influence across the entire study period (McArdle, 2009; Willoughby et al., 2012). For example, in an RPPF design where the treatment effect is hypothesized to occur immediately post-intervention but fade by follow-up, LCMs can estimate these discrete changes directly. This makes them well-aligned with the practical questions guiding most clinical and behavioral trials, where investigators seek to understand not only whether change occurred, but when. By modeling the change process itself, rather than simply comparing timepoint means or estimating a global slope, LCMs offer a nuanced and theoretically grounded approach (Mara et al., 2012; Mun et al., 2009).

LCMs retain many of the strengths of LGCMs and LMMs, including the ability to incorporate time-invariant and time-varying covariates, model individual variability, and integrate into mediation or multivariate frameworks. Moreover, anchoring the latent intercept at different timepoints (e.g., pretest, posttest, or follow-up) allows LCMs to align the model structure with the temporal focus of the research question (Willoughby et al., 2007). This flexibility makes LCMs an appealing option for applied researchers, especially when used in tandem with graphical outputs and model fit indices to evaluate competing hypotheses.

Although the example analysis of the STAR trial presented below contrasts a LCM with a LGCM and a LMM, these comparisons reflect differences in parameterization rather than wholly distinct model families. In fact, LGCMs can approximate LCM-like behavior when spline or piecewise specifications are used (Curran et al., 2010), and LMMs can produce similar results with appropriate coding of time and interaction terms. However, LCMs make these contrasts more explicit and interpretable within the SEM framework, which may benefit applied researchers aiming to evaluate change across specific, theoretically meaningful timepoints, such as those inherent in RPPF designs.

LCMs have been increasingly applied in developmental and intervention research, including studies of executive function in early childhood (Willoughby et al., 2012), longitudinal change in fluid intelligence (Kievit et al., 2018), and cognitive-behavioral intervention outcomes among patients with Alzheimer’s disease (Werheid et al., 2015). These examples illustrate the growing utility of LCMs in capturing discrete, phase-specific changes while leveraging the flexibility of SEM. In summary, LCMs offer a conceptually intuitive and statistically rigorous approach to modeling change in RPPF designs. By estimating latent difference scores at each phase, the ability to account for measurement error, and enabling covariate inclusion and hypothesis testing within a single model, LCMs help researchers evaluate not only whether change occurred, but how, when, and for whom.

### Comparing model structures

Below we present simplified representations of how each model structure estimates change and treatment effects in RPPF designs:

**ANCOVA:**

$$Y_{post} = \beta_{0} + \beta_{1} \cdot Group + \beta_{2} \cdot Y_{pre} + \varepsilon$$  

This model adjusts posttest scores for pretest values using regression, typically analyzed separately for each timepoint.

**LMM:**

$$\begin{aligned} Y_{{it}} = & \beta _{0} + \beta _{1} \cdot {\mathrm{Time}}_{t} + \beta _{2} \cdot {\mathrm{Group}}_{i} \\ & + \beta _{3} \cdot ({\mathrm{Time}}_{t} \times {\mathrm{Group}}_{i} ) \\ & + u_{{0i}} [ + u_{{1i}} \cdot {\mathrm{Time}}_{t} ] + \varepsilon _{{it}} \\ \end{aligned}$$  

This model estimates change over time with a random intercept (and an optional random slope) and an interaction term to test group differences in trajectories.

**LGCM:**

$$Y_{t} = \eta_{0} + \eta_{1} \cdot \lambda_{t} + \varepsilon_{t}$$  

$$\eta_{1} \leftarrow \gamma \cdot {\mathrm{Group}}{}$$  

Where η_0_ and η_1_ are latent intercept and slope factors, and λ_*t*_ are loadings representing time. Group differences are tested by regressing the slope on Group.

**LCM:**

Define adjacent latent changes Δ_*it*_ for each segment (i.e., the change in the outcome from *t*-1 to *t*; e.g., pretest → posttest, posttest → follow-up 1, follow-up 1 → follow-up 2, follow-up 2 → follow-up 3). The model, with baseline adjustment, is

$$ Y_{{it}} = Y_{{i,t - 1}} + \Delta _{{it}} + b_{t} Y_{{i0}} (t \ge 1), $$

and the change equation is

$$\Delta_{it} = \mu_{t} + \beta_{t} \cdot {\mathrm{Group}}_{i} + u_{it} (t = 1, \ldots )$$     

Here, β_*t*_ tests the between-group difference in mean change in the outcome for each segment. Note that this is a pure-change specification with no proportional-to-level component (i.e., γ_*t*_ = 0 for all *t*). Baseline enters the model on the observed post-baseline outcomes via *b*_*t*_*Y*_*i*0_ (ANCOVA-style baseline adjustment).

These formulations highlight key differences in how each model is typically used to conceptualize and estimate change, from adjusting for baseline (ANCOVA), modeling overall trajectories (LMM/LGCM), to capturing discrete changes over time (LCM).

### Missing data handling in RPPF designs

Missing data are common in RPPF trials due to missed visits and attrition, and how they are handled materially affects bias, precision, and interpretation. A practical default is the missing at random (MAR) assumption, which is often reasonable in trials when baseline outcome and other prognostic variables are included in the model (Enders, 2010; Little, 2013).

For SEM models (LGCM, LCM), parameters can be estimated using full-information maximum likelihood (FIML) with robust standard errors (MLR). FIML uses each participant’s observed data without listwise deletion or ad hoc imputation and yields consistent, efficient estimates when MAR holds. For LMMs, maximum likelihood/restricted maximum likelihood (ML/REML) likewise uses all available observations under MAR when the mean and covariance structure are correctly specified. By contrast, a single-timepoint ANCOVA is not inherently robust to missingness and often defaults to complete cases unless paired with a principled approach such as multiple imputation (MI) (Enders, 2010; Graham, 2009).

Two practical steps help make MAR more plausible: include strong predictors of the outcome and of missingness (such as the baseline outcome, site, and pre-specified covariates) in the analysis; and, where relevant, treat such variables as auxiliaries in SEM or include them in the imputation model for MI. These choices retain sample size, preserve power, and reduce bias relative to complete-case analyses when missingness relates to observed characteristics.

### The STAR trial

The Supporting Treatment Adherence Regimens (STAR) trial was a randomized clinical trial designed to assess the efficacy of a family-tailored behavioral intervention aimed at improving adherence to anti-seizure medications (ASMs) in young children (2–12 years old) with newly diagnosed epilepsy (Modi et al., 2021). The trial compared the STAR intervention, which used problem-solving strategies to address family-specific adherence barriers, to an education-only (EO) attention control group. The primary goal was to improve ASM adherence, with secondary outcomes examining seizure control and health-related quality of life (HRQOL).

Participants were recruited from a pediatric epilepsy center within seven months of their epilepsy diagnosis. Inclusion criteria required children to be on a single ASM and demonstrate suboptimal adherence (< 95%) during a baseline monitoring period. Eligible participants were stratified by baseline adherence and randomized into either STAR or EO. Both groups included eight sessions over four months (six in-person and two phone sessions), with assessments conducted at baseline and then at 3 months (post-intervention), as well as 6 and 12 months (2 follow-ups). Adherence (primary outcome) was measured electronically using medication event monitoring systems, averaged across each month as the number of doses taken divided by the number of doses prescribed and ranged from 0–100%, thus all coefficients reported below are interpretable as percentage points. A descriptive summary of the primary outcome by group and timepoint is provided in Table 1.

### STAR trial results analyzed by ANCOVA

ANCOVA models indicated no significant differences in adherence at posttest (b = 3.99, se = 3.68, *p* = 0.28, 95% CI: −3.30, 11.29) or at the 3-month follow-up (b = 4.55, se = 5.41, *p* = 0.40, 95% CI:− 6.18, 15.28). However, significant differences emerged at later timepoints. At the 6-month follow-up, there was a significant difference in adherence between the intervention groups (b = 9.56 percentage points, se = 4.65, *p* = 0.04, 95% CI: 0.34, 18.77), which was further pronounced at the 12-month follow-up (b = 15.10 percentage points, se = 5.32, *p* = 0.006, 95% CI: 4.50, 25.68).

### STAR trial results analyzed by LMM

**Fig. 1:**
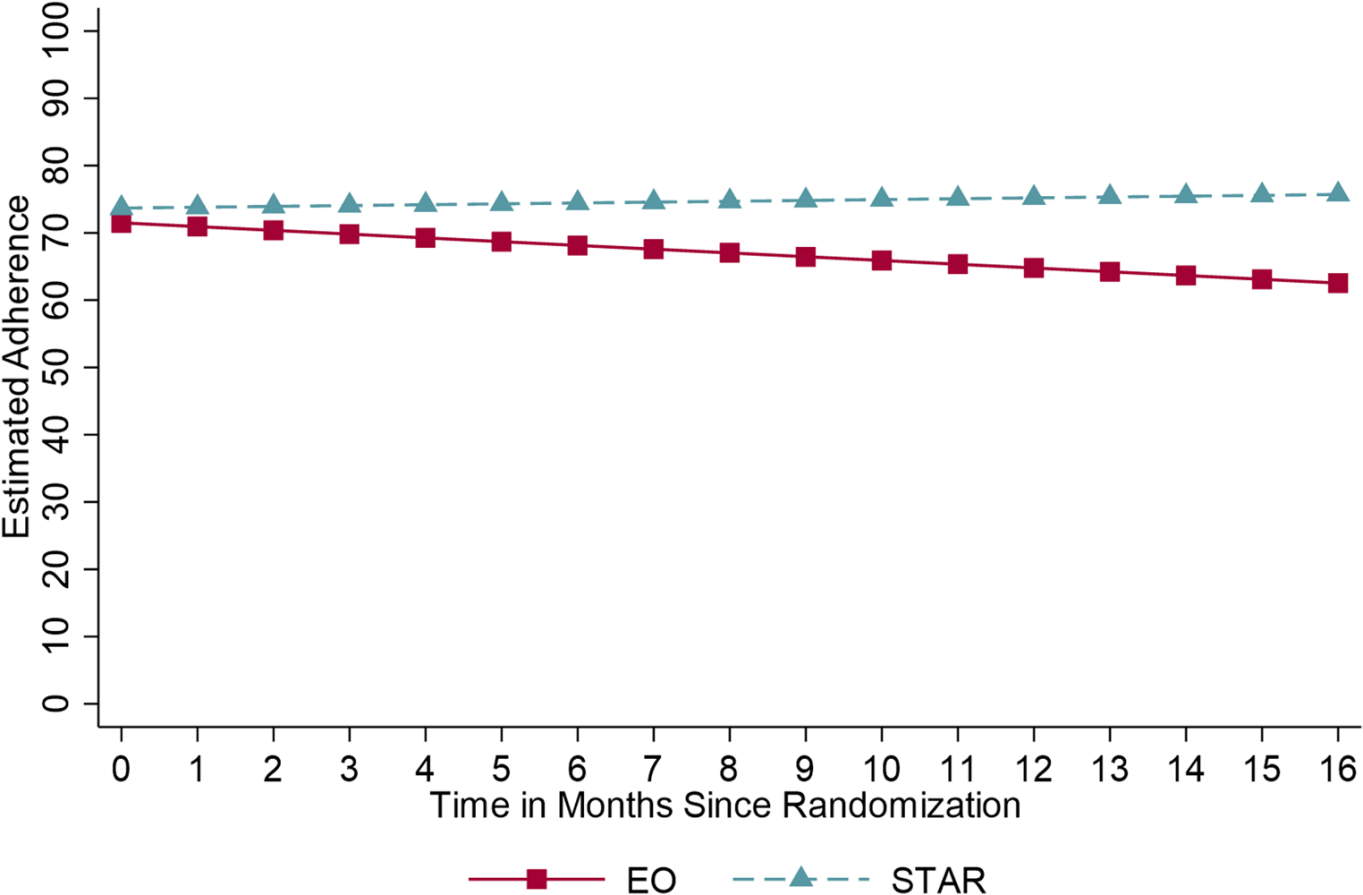
Estimated intervention group trajectories from the longitudinal mixed-effects model of the STAR trial data

The results from the longitudinal mixed-effects model indicated that the average intervention group trajectories of adherence from baseline to the 12-month follow-up were not significantly different (See Fig. 1). Specifically, the group-by-time interaction was not significant (b = 0.69, se = 0.38, *p* = 0.07), suggesting that the intervention groups did not significantly differ in the average adherence trajectories. Of note, there was no evidence of a significant non-linear effect.

### STAR trial results analyzed by LGCM

**Fig. 2:**
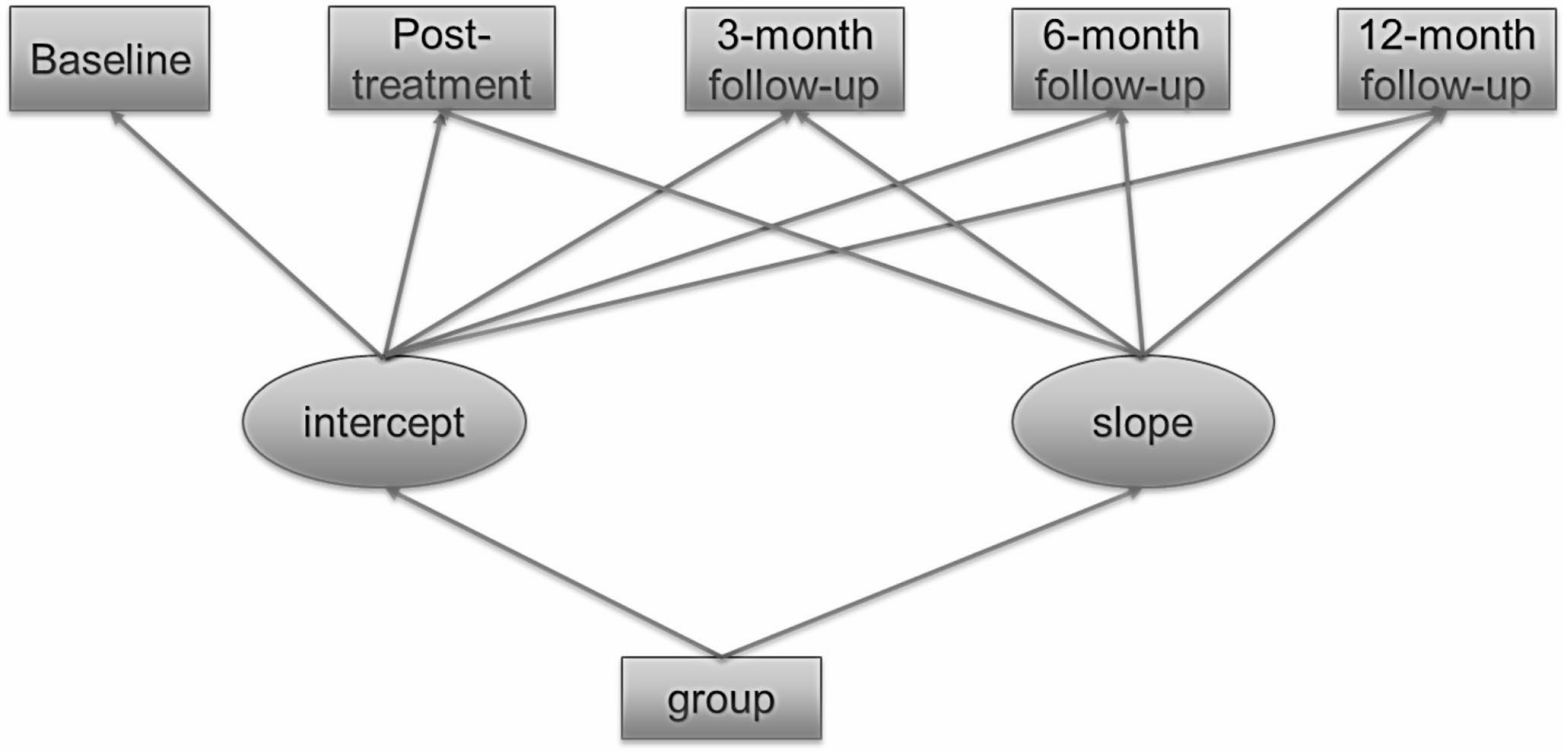
Conceptual model for the latent growth curve model of the STAR trial

The conceptual model of the LGCM is presented in Fig. 2. It is worth noting here that in order to facilitate direct estimation of group differences in change and ensure comparability across ANCOVA, LMM, LGCM, and LCM models, group was modeled as a covariate in all analyses. This parameterization targets differences in expected change while implicitly assuming equality of residual and change-score (co)variances across groups. However, a multigroup SEM framework could also be used to examine differences in structural parameters across intervention groups and may be particularly useful in future research aiming to test parameter equality across groups (Bollen & Curran, 2006). When hypotheses concern equality of variances/covariances (e.g., residual variances, change-variance, or cross-time residual covariances), a multi-group SEM framework is more appropriate, because it enables direct tests across a broader set of parameters.

The results from the latent growth curve model revealed that the rates of change (slopes) of the intervention groups were significantly different (b = 3.19, se = 1.30, *p* = 0.01), highlighting a modest (~ 3 percentage points) difference in adherence trajectories between the intervention groups. However, model fit indices suggested room for improvement, with high RMSEA (0.22) and suboptimal CFI (0.57), TLI (0.51), and SRMR (0.14), and thus parameter interpretations should be viewed cautiously given this misfit. A single linear-slope LGCM was fitted for comparability and parsimony, thus the suboptimal fit likely reflects a researcher specification resulting in misalignment between the assumed linear growth structure and the actual pattern of change. Given the poor fit of the single-slope LGCM in this instance, it would be prudent to test additional forms of change such as non-linear, piece-wise models, or estimate splines.

### STAR trial results analyzed by LCM

**Fig. 3:**
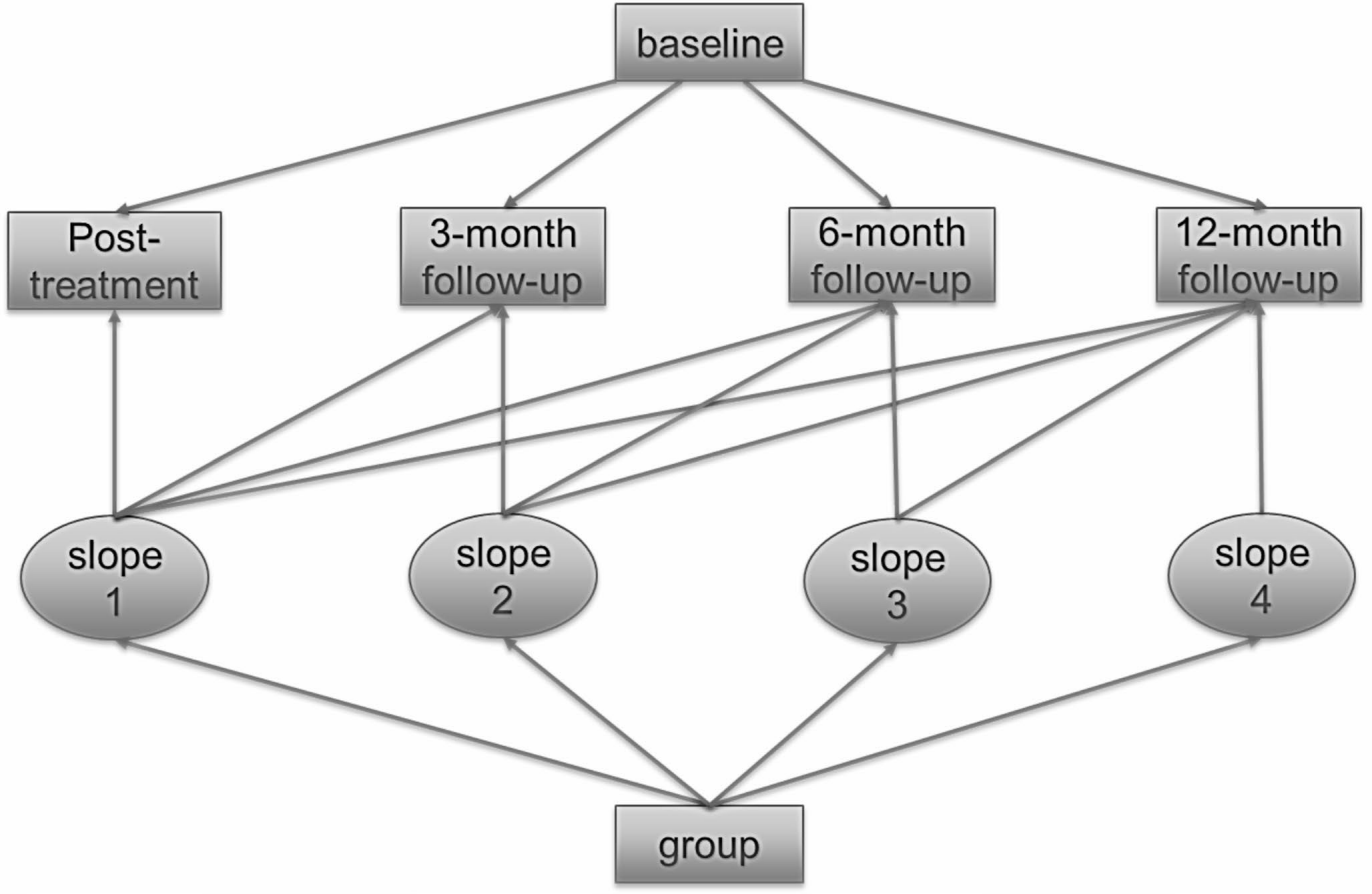
Conceptual model for latent change model of the STAR trial

The conceptual model of the LCM is presented in Fig. 3, where Slope 1 is the amount of change from pretest to posttest, Slope 2 is the amount of change from posttest to 3-month follow-up, Slope 3 is the amount of change from 3-month follow-up to 6-month follow-up, and Slope 4 is the amount of change from 6-month follow-up to 12-month follow-up. Thus, the regression of slope 1 on group can be interpreted as a test of intervention group differences in change from pretest to posttest, and the regression of Slopes 2–4 on group can be interpreted as a test of intervention group differences in change between adjacent follow-ups. The code to implement this model in Mplus is presented in the Appendix.

Model fit indices indicated an excellent fit to the data, with RMSEA = 0.06, CFI = 1.00, TLI = 0.96, and SRMR = 0.02. The LCM model results showed no significant difference in the intervention groups from pretest to posttest (b = 3.99, se = 3.63, *p* = 0.27), posttest to the 3-month follow-up (b = −2.09, se = 3.35, *p* = 0.53), or 3-month follow-up to the 6-month follow-up (b = 6.82, se = 5.54, *p* = 0.22). However, there was a significant difference between the intervention groups in the change from 6-month follow-up to the 12-month follow-up (b = 7.04, se = 3.00, *p* = 0.02), indicating divergence in adherence (~ 7 percentage points) between the intervention groups in the final follow-up interval (6 months through 12 months follow-up).

### Step-by-Step guide: latent change score modeling for RPPF trials

The goal in this section is to provide researchers with the key decision points needed to estimate discrete changes between adjacent timepoints in trial data, then test intervention group differences in those changes. It is assumed that one outcome is measured at pretest, posttest, and follow-ups and that your Group variable is coded 0 = control, 1 = intervention.

1. *Define the segments you care about*
  List the adjacent change segments you will estimate (e.g., pre → post, post → 3-month, 3-month follow-up → 6-month follow-up, etc.). Include only segments that align with your design and hypotheses (e.g., the primary posttest window plus clinically meaningful follow-ups).
2. *Build a latent change factor for each segment*
  Create one latent variable per segment that represents after minus before for that pair of timepoints. Each observed timepoint equals a baseline level plus the sum of all prior changes (so changes accumulate naturally). Either: (a) define each change explicitly as “after minus before,” or (b) use a simple step setup where each change “turns on” at its segment and stays on thereafter (similar to the code presented in the appendix). Both yield the same interpretation (mean change for that segment).
3. *Estimate group differences in each change*
  Regress each latent change on Group. The coefficient is the between-group difference in mean change for that segment (intervention minus control). If you need to compare variances/covariances across groups (not just means), use a multi-group specification instead of (or in addition to) modeling group as a covariate.
4. *Residuals*
  In the Appendix model, a pure-change specification is used: residual variances at post-baseline timepoints are fixed to 0 and residual correlations across time are constrained to 0. This simplifies interpretation such that post-baseline variability is attributed to the latent change factors, but assumes no residual autocorrelation beyond the change process. Because the same outcome is administered repeatedly, an applied alternative is to estimate residual variances and allow small adjacent residual correlations (e.g., post with follow-up 1, follow-up 1 with follow-up 2) if diagnostics indicate leftover dependence. Start with adjacent pairs to retain parsimony and expand only if fit indices, modification indices, or residuals justify it.
5. *Decide whether change varies across people*
  In the appendix, the variances (and, by default, covariances) of the latent change factors (s1–s4) are estimated, allowing person-to-person variability in each segment’s change. Because Group is included as a covariate, those variances are not compared across groups. Testing variance (non)invariance would require a multi-group model. For a purely average-change model, fix change variances to 0. If the model estimates change variance as negative, (a) fix it to a small positive value, (b) constrain equal across adjacent segments, or (c) fix to 0 if an average-change model is acceptable.
6. *Handling missing data and assessing model fit*
  Use full-information maximum likelihood (FIML) with robust standard errors so participants with partial data are retained. If fit is marginal, first reconsider residual correlations (Step 4), your assumption about variation in change (Step 5), and whether your “segments” match the design (Step 1).
7. *Pre-specify covariates*
  Include only covariates that were pre-specified and are clearly prognostic (e.g., baseline outcome, site, key demographics). Avoid post-randomization variables. Add the same covariates to every change factor to keep interpretation consistent.
8. *Note about baseline*
  The model estimated in the appendix adjusted for baseline using an ANCOVA-style specification in which each post-baseline observed score was regressed on the pretest. This yields baseline-conditional estimates of discrete change and group differences, controls regression-to-the-mean, and aligns with common practice across SEM, LMM, and trial reporting. If the model struggles (non-convergence or misfit not solved by residual/variance tweaks), here are some options to consider:
  - Free baseline slopes across timepoints: Don’t force one baseline effect to apply to post and all follow-ups. Let each timepoint have its own baseline slope. If theory suggests similarity, test equality after freeing.
  - Check moderation of baseline by group: If baseline relates to outcomes differently across.groups, add a Group × Baseline term (or use a multi-group LCM). Forcing a common baseline slope can mask misfit.
  - Relax the “pure-change” constraints slightly: If you fixed post-baseline residual variances to 0, allow small residual variances and/or adjacent residual correlations (posttest ↔ follow-up 1, follow-up 1 ↔ follow-up 2).
  - Move where baseline enters the model (re-parameterize): If “baseline → observed scores” is unstable, try “baseline → latent change factors” instead.
  - Strengthen the baseline measurement (if you can): If multiple baseline indicators exist, form a latent baseline factor to reduce measurement error and stabilize the adjustment. Note that this can be applied to all observed score indicators at all timepoints.

## Discussion

This tutorial highlights the strengths of latent change models (LCMs) in analyzing longitudinal data from behavioral intervention trials. While ANCOVAs, LMMs, and LGCMs remain powerful analytical approaches, LCMs offer several strengths when applied to RPPF designs:

- *Estimates of discrete changes between timepoints*: LCMs allow for granular understanding of how interventions influence change in the outcome at specific intervals of the trial, providing richer insights into intervention effects.
- *Incorporation of all longitudinal data into a single model*: Each discrete change segment is estimated jointly, leveraging the full dataset rather than relying on piecemeal analysis and improving precision.
- *Uses all available data under MAR*: FIML addresses incomplete data, such that participants with partial follow-ups are retained, and auxiliary predictors can be included to support the MAR assumption.
- *Improved power through baseline covariate adjustment*: Covarying baseline controls initial differences and reduces variance, boosting the ability to detect meaningful effects.
- *Flexibility within the SEM framework*: Researchers can incorporate measurement models, add time-varying or time-invariant covariates, specify parallel-process or mediation structures, conduct multi-group and invariance testing, and relax or re-specify residual/variance constraints when diagnostics warrant – all within the same LCM.

Collectively, these strengths position LCMs as a practical, information-efficient choice for RPPF trials, where the primary research questions often center on between-group differences in discrete, interval-specific changes.

Participants in the STAR intervention group demonstrated slight improvements in their adherence and maintained overall higher adherence levels throughout the study. In contrast, adherence rates in the EO group steadily declined, consistent with patterns commonly observed among newly diagnosed patients, where adherence wanes as initial motivation diminishes. These findings suggest that the STAR intervention's primary benefit lies in stabilizing adherence levels, preventing the typical decline seen over time rather than substantially improving adherence rates. This distinction is critical for understanding the intervention's practical implications and designing future enhancements.

The STAR Trial results were analyzed using four approaches: ANCOVA, LMM, LGCM, and LCM, offering complementary perspectives on intervention group differences over time. While the findings were generally consistent, notable divergences emerged in identifying significant differences at specific timepoints. Specifically, both ANCOVA and LCM analyses agreed that there were no significant differences between the intervention groups at posttest or the 3-month follow-up, and both methods identified significant differences by the 12-month follow-up. However, the ANCOVA and LCM models diverged in their findings for the 6-month follow-up. While ANCOVA identified a significant group difference at this timepoint, the LCM did not, as the overall estimated intervention effects were smaller in the LCM than those estimates in the ANCOVA models. The LMM approach, on the other hand, revealed no significant overall group-by-time interaction and suggested that adherence trajectories across the groups were not significantly different over the 12-month period. In contrast, the LGCM highlighted a significant group difference in the slopes of adherence trajectories, suggesting modest but meaningful differences in adherence over time. Model fit indices varied across the two SEM-based approaches. The single-slope LGCM fit poorly, indicating a mismatch between a strictly linear trajectory and the observed pattern and not a limitation of the LGCM approach (or SEM) per se. Although trajectory models are often run with a single slope by default, they readily accommodate nonlinearity via piecewise slopes, spline knots (e.g., at post and 6 months), or a latent-basis specification with free loadings. In line with this, the LCM’s adjacent-change parameterization captured the interval-specific shifts and fit well. The ‘poor fit’ conclusion for the LGCM of the STAR data therefore applies only to the chosen linear specification; alternative LGCM parameterizations could also represent these data.

These differences underscore the importance of carefully considering the study hypotheses and the clinical or theoretical implications of specific timepoints when interpreting trial results and drawing overall conclusions. Each analytical approach (ANCOVA, LMM, LGCM, and LCM) answers slightly different questions about the trial data, emphasizing distinct aspects of intervention effects. For instance, ANCOVA provides a straightforward assessment of group differences at specific timepoints, while LMM evaluates overall trajectories and the interaction with intervention group over time. LGCM focuses on modeling growth patterns and capturing variability in trajectories, whereas LCM offers a more discrete perspective by breaking the timeline into distinct segments to examine changes across specific intervals. Researchers must align their choice of analytical technique with the primary goals of the study, determining whether the focus is on pinpointing differences at discrete timepoints, understanding overall trends, or evaluating changes during critical periods. Such careful alignment ensures that the selected method provides insights that are not only statistically robust but also clinically and theoretically meaningful for the trial’s objectives.

Researchers are encouraged to consider the use of LCMs as an efficient and robust method for analyzing data from RPPF designs. The ability of LCMs to estimate discrete changes, accommodate missing data, and incorporate baseline covariate adjustment positions them as a particularly powerful and efficient strategy for assessing intervention effects in behavioral intervention trials. While not advocating for the replacement of ANCOVAs, LMMs, or LGCMs as analytical tools for RPPF designs, as these methods have notable strengths in analyzing data from RPPF trials, this paper highlights how LCMs can complement these approaches and, in some cases, provide a potentially more efficient means of answering the research questions specific to RPPF designs in evaluating both short-term and sustained intervention effects.

## Supplementary Information

Below is the link to the electronic supplementary material.

Supplementary Material 1

## Footnotes

1 Note: SEM accounts for measurement error when outcomes are modeled as latent variables with multiple indicators. In our tutorial example presented below, we use single-indicator observed composites and a pure-change specification (post-baseline residual variances fixed to 0), so measurement error at post-baseline timepoints is not estimated separately.
