# Supplementary material for: Methods for analyzing longitudinal data from randomized pretest-posttest-follow-up trials in behavioral research: a practical guide to latent change models
Source: J Behav Med. 2025 Sep 9;49(2):286–97. doi: 10.1007/s10865-025-00600-y (PMC13253591; doi:10.1007/s10865-025-00600-y)
Supplement: Supplementary file 1 — Supplementary Material 1 [file 10865_2025_600_MOESM1_ESM.docx]

**Appendix**

TITLE: Latent Change Score Model STAR data

DATA:

FILE = yourdata.dat;

VARIABLE:

NAMES = id group adh0 adh5 adh7 adh10 adh16;

USEVARIABLES = group adh0 adh5 adh7 adh10 adh16;

MODEL:

! regress post-baseline observed scores on pretest

adh5 adh7 adh10 adh16 on adh0;

! Latent change score defined

s1 | adh5@1 adh7@1 adh10@1 adh16@1;

s2 | adh7@1 adh10@1 adh16@1;

s3 | adh10@1 adh16@1;

s4 | adh16@1;

! Regress latent change scores on condition (group)

s1 s2 s3 s4 ON group;

! Fix residual variances of observed scores to zero

adh5@0;

adh7@0;

adh10@0;

adh16@0;

! Explicitly estimate mean of baseline to invoke FIML

[adh0];
